# Understanding the role of incentives for achieving and sustaining viral suppression: A qualitative sub-study of a financial incentives trial in Uganda

**DOI:** 10.1371/journal.pone.0270180

**Published:** 2022-06-24

**Authors:** Carol S. Camlin, Kara Marson, Alex Ndyabakira, Monica Getahun, Devy Emperador, Ambrose Byamukama, Dalsone Kwarisiima, Harsha Thirumurthy, Gabriel Chamie

**Affiliations:** 1 Department of Obstetrics, Gynecology & Reproductive Sciences, University of California San Francisco, San Francisco, California, United States of America; 2 Department of Medicine, University of California San Francisco, San Francisco, California, United States of America; 3 Infectious Diseases Research Collaboration, Kampala, Uganda; 4 Perelman School of Medicine, University of Pennsylvania, Philadelphia, Pennsylvania, United States of America; Nnamdi Azikiwe University, NIGERIA

## Abstract

**Background:**

Viral suppression among people living with HIV (PLHIV) is essential for protecting health and preventing HIV transmission, yet globally, rates of viral suppression are sub-optimal. Interventions to improve HIV prevention and care cascade outcomes remain vital. Financial incentives hold promise for improving these outcomes, yet to date, clinical trial results have been mixed.

**Methods:**

This qualitative sub-study, embedded in a trial (NCT02890459) in Uganda to test whether incentives are effective for achieving viral suppression in PLHIV, sought to enhance our understanding of the factors that influence this outcome. Forty-nine (n = 49) PLHIV, purposely sampled to balance across gender, study arm, and viral suppression status, were interviewed to explore barriers and motivations for care engagement, adherence, and viral suppression, and attributions for decision-making, including perceived influence of incentives on behaviors.

**Results:**

While many participants with undetectable viral load (VL) who received incentives said the incentives motivated their ART adherence, others expressed intrinsic motivation for adherence. All felt that incentives reduced burdens of transport costs, lost income due to time spent away from work, and food insecurity. Incentives may have activated attention and memory for some, as excitement about anticipating incentives helped them adhere to medication schedules. In comparison, participants who were randomized to receive incentives but had detectable VL faced a wider range, complexity and severity of challenges to care engagement. Notably, their narratives included more accounts of poor treatment in clinics, food insecurity, and severe forms of stigma. With or without incentives, adherence was reinforced through experiencing restored health due to ART, social support (especially from partners), and good quality counseling and clinical care.

**Conclusions:**

In considering why incentives sometimes fail to achieve behavior change, it may be helpful to attend to the full set of factors- psychological, interpersonal, social and structural- that militate against the behavior change required to achieve behavioral outcomes. To be effective, incentives may need to be combined with other interventions to address the spectrum of barriers to care engagement.

## Introduction

Viral suppression among people living with HIV (PLHIV) is essential for protecting health and preventing HIV transmission. Despite substantial progress in scaling-up antiretroviral therapy (ART), an estimated 59% of PLHIV globally, including ~65% of PLHIV in eastern and southern Africa, remain virally unsuppressed as of 2019 [1]. As many countries aim to meet the UNAIDS 95-95-95 target to achieve HIV epidemic control, there is a vital need for interventions to improve outcomes along the HIV prevention and care cascades [2].

The use of monetary and non-monetary incentives to promote behavior change builds upon behavioral economics and contingency management principles [3], and a large body of research showing that incentives have led to a range of positive health outcomes [4–6]. The potential of incentives for improving prevention and care cascade outcomes has been examined in several HIV clinical trials with mixed results. In sub-Saharan Africa (SSA), incentives have been shown to be effective in promoting uptake of HIV testing [7] and medical male circumcision [8], but were not found to improve linkage to care [9, 10] or antiretroviral therapy initiation [9]. However, two studies in the region found positive effects of incentives—conditional cash [10, 11] and food assistance [11]—on ART adherence, and one found conditional cash incentives to be effective in retaining pregnant women in care [12]. In the United States, two large trials showed beneficial effects of financial incentives on improving viral suppression [10, 13]. The present qualitative sub-study was embedded in the first clinical trial (NCT02890459) conducted in SSA to test whether financial incentives could increase achievement and maintenance of viral suppression among PLHIV [14]. We found that financial incentives did not increase the likelihood of viral suppression at 24 weeks.

We have commented previously that higher than expected baseline viral suppression levels, and provision of viral load results and counselling to all participants may in part explain why incentives were not effective in increasing viral suppression [14]. However, subgroup analyses showing that participants unsuppressed at baseline did not respond to financial incentives suggest that alternative explanations, as well as approaches to address them, are needed. This sub-study was designed to enhance our understanding of the factors, particularly in the Ugandan context, that influence whether and how financial incentives can be effective for both achieving and sustaining viral suppression. We sought to understand the barriers and motivators for care engagement and achievement of viral suppression, with a focus on participants’ perceptions of the financial incentives and their role in influencing behaviors and outcomes.

## Methods

### Study design

This qualitative study was embedded in the ‘Innovative Incentive Strategies for Sustainable HIV Testing and Antiretroviral Treatment’ trial, a 2-arm randomized trial conducted among 400 PLHIV in rural southwestern Uganda, from June 2016 through May 2018, to study the effectiveness of financial incentives for both achieving and sustaining viral suppression [7]. The intervention group received escalating financial incentives (from US$4 to $12.5) for a period of 24 weeks, with rewards *contingent on having an undetectable viral load* at 6, 12, and 24 weeks. The procedures, briefly, were as follows: HIV-positive men and women were identified from multi-disease community health campaigns offered in the study communities in June and July 2016. To meet enrollment targets for the trial, additional HIV-positive adults who lived within the study communities were recruited and enrolled between October 2016 and May 2017, as previously described. Trial participants were offered escalating incentive amounts upon achievement and maintenance of virological suppression, and followed up for one year post-randomization. After the follow up was completed, trial outcome data were used to conduct purposive sampling for the qualitative study. To attain heterogeneity across purposive sampling categories for this study, 49 participants were systematically selected from lists of participants grouped by study arm, gender, and either success or failure at achieving and maintaining HIV viral suppression during the follow up period.

### Data collection and analysis

In-depth semi-structured interviews (IDIs) were conducted using guides tailored to the study arm and HIV viral suppression groupings. Two qualitative researchers conducted interviews in the local language, Runyankole, at the participant’s preferred location. We developed four versions of the in-depth interview guide to collect data among participants: two versions for the control, and two for the intervention (i.e. randomized to receive incentives), and within each arm, a version each for participants with suppressed VL and detectable VL, respectively. All guides included questions to elicit participants’ perceptions, attitudes and preferences related to ART, including motivations, barriers, and psychosocial and contextual factors that influence HIV care and ART adherence decisions. Additionally, guides for those not in care included questions on barriers to care, while guides for those receiving incentives included questions on the role of incentives in HIV care decision making. Audio recordings were translated, transcribed into English, and deductively and inductively coded. An initial coding framework was developed on the basis of the theory-informed domains of inquiry for interview guides; these codes were applied using qualitative analysis software by a four-person team under the supervision of the lead investigator. The coding framework was iteratively refined during data collection and analysis, following review and discussion of empirical findings from the data. Coded excerpts were extracted, reviewed and analyzed for further reductions, with a final stage of analysis aimed at ascertaining divergence or convergence of emergent themes across the purposive sampling categories of study arm, gender, and either success or failure at achieving and maintaining viral suppression. The emergent themes with supportive evidence are presented below; transcript excerpts identify individuals by their study ID, age and gender.

### Ethical approvals

All participants provided informed consent to participate. The study was approved by the institutional review boards of Makerere University *(SOMREC 2015–138)*, the Uganda National Council for Science and Technology *(SS3980)*, and the University of California, San Francisco *(15–16876)* (the latter of which served as the institutional review board of record for investigators from the University of Pennsylvania).

## Results

We summarize emergent themes related to factors that led to, or inhibited, starting and adhering to ART, and staying engaged in care. We compare these themes across categories of participants grouped by their study-defined ‘success’ (viral suppression at 24 weeks after intervention exposure) or ‘failure’, gender, and study arm (randomized to receive incentives, or not). To focus on the key question of the role of incentives in influencing outcomes, we highlight findings and variations by analytical categories of viral suppression compared to detectable VL among the PLHIV randomized to receive incentives. For brevity’s sake, findings in the control arm within these categories are briefly summarized, with a focus on discernable divergence from themes in the narratives of PLHIV, grouped by viral suppression status, in the intervention arm.

### Experiences and attributes of virally suppressed PLHIV

#### Women

Women who received incentives and had undetectable viral loads at 24 weeks after intervention reported overcoming multiple barriers to ART initiation, adherence, and care engagement, including denial, internalized stigma, and anticipated stigma:

“I did not start taking drugs immediately because I could not believe that I was HIV positive.”

(11716, woman, age 39)

“At times I am so depressed, that the only solution is death… though after some time, I think otherwise.”

(11494, woman, age 54)

“HIV is a secret which people cannot easily disclose or talk about. […] I feared that people might know [my HIV status] because of these cars [study vehicles] that come in the village looking for me.”

(16297, woman, age 46)

These women had overcome fears of the side effects of ART, and actual experiences of side effects early in treatment: “At first I thought it [ART] would make me dizzy and change the way I look physically…. I knew it would kill me, but I did not die.” (16663, woman, age 54)

There were instances of difficulties with the health system, with one woman having experienced multiple false negative tests and another reporting verbal abuse and expired tablets at a health facility: “I refused to go to the parish center, because I was told the health worker abuses patients. When I got medication at that place, she gave me expired tablets.” (11494, woman, age 54). Women also described having had challenges with paying for transport to clinics, particularly if clinics are far from home: “I have now shifted to a far place from the clinic and getting money for transport might be hard.” (16004, woman, age 27). To save money, those living closer to clinics often walk, but this presented difficulty for older women: “Walking is a challenge. One side of my body usually gets pain. I have to sit on the way, because of walking a long distance to the health centre to pick medication.” (11494, woman, age 54). Yet, despite these obstacles, women opted to start ART, adhering successfully, and described actively taking on treatment:

“I did request for the ARVs myself. […] I was not afraid because even those who are not positive meet problems and other diseases too. I loved myself and began ARVs so that my viral load reduces as we were told by the counselors.”

(10537, woman, age 36)

Others described having simply deferred to providers who told them it was time to start, rather than consciously making a decision about it themselves. “Doctors are the ones who decided that I should start on ARVs because of poor health which I had. It was not my own will to start.” (11716, woman, age 39). Yet overwhelmingly, women in this group expressed a strong intrinsic motivation to stay engaged in care and treatment. For some, experiencing the restoration of their health, enabling them to work and feel ‘normal’ motivated their adherence:

“After taking the medication all the fevers stopped and I never got any other types of illness.”

(11494, woman, age 54)

“…I do my work normally. I used to develop fever in the evening, but when I started taking ARVs, it stopped.”

(16663, woman, age 54)

“Before I started treatment, I used to be too weak but since I began treatment, I am strong and that is why I have to remain true to treatment. If I had dropped, I would have suffered with the pregnancy. This made me love myself more, just like any normal person.”

(10537, woman, age 36)

Women who were virally suppressed described receiving social support from spouses, friends or family members: “even when I do not come for treatment he [her husband] brings me the drugs, and I also do the same in case he is unable to make it. So we now take the treatment at the same time.” (10537, woman, age 36) Yet even women without supportive spouses, in this group, were strongly motivated to persist in care-seeking and adherence to ART; notably, in these instances the spouses did not actively oppose their care engagement:

[*Interviewer*: *Do you discuss with your husband about HIV care and treatment*?] “No, because I don’t stay with him, and we don’t know each other’s status.”

(16373, woman, age 33)

“I try sometimes to discuss [HIV care] with him, but he shows that he is not interested in such kind of discussion.”

(16663, woman, age 54)

Women in this group were overall knowledgeable about the links between ART adherence and viral load, and credited viral load counseling for reinforcing their adherence:

“It motivated me a lot and I even changed my schedule so that I am able not to miss my exact hour and minute of taking ARVs.”

(11716, woman, age 39)

“I was extremely happy… I knew that the drugs which I am taking works and I have rightly followed doctor’s instructions.”

(16004, woman, age 27)

Women in this group expressed delight and gratitude about having received financial incentives. Some felt personally cared for, to receive an incentive:

“I felt happy because it is rare for one to be given an incentive, and yet it is your own life. I felt very happy for being loved.”

(10537, woman, age 36)

The incentive helped to motivate continued adherence, and the escalating structure was reported to be especially encouraging:

“I was motivated to take my medication without missing so that the amount would increase.”

(11494, woman, age 54)

Family members who knew about incentives encouraged some participants to adhere to ART for the sake of the incentive: “He encouraged me to stick on ARVs so that I can receive the prize.” (16004, woman, age 27). Where the incentives were especially helpful were that they activated attention and memory, as women described their excitement about anticipating the incentives as a factor in helping them to keep to their schedule for medication adherence:

“Before I used to forget to take my dose, and even I would not be having specific time of swallowing my drugs, but since I was told about the incentive, I became strict and followed my schedule without missing.”

(11716, woman, age 39)

“It motivated me a lot to take my medications on schedule without missing and also keeping the appointments without fail. Even if I don’t have money, I have to borrow it so that I can’t miss the clinic day…. they said that each time they come and find when I have achieved viral suppression, they will keep on adding on the value of the prize.”

(16004, woman, age 27).

Incentives were reported to reduce the challenges of keeping to clinic appointments due to lack of money for transport: “It motivated me to continue taking ARVs and it also helped me to solve transport related challenges.” (16297, woman, age 46). It also allowed women to not have to make difficult decisions about whether to use money for transport to clinic, or food:

“At the time I felt like eating meat, but I had no money. So when they gave me money… I walked [home from the clinic], bought meat and ate it. And felt so happy and encouraged to continue taking my medication.”

(11494, woman, age 54)

Some women said they would have been likely to adhere to medications even if they had not received the incentives:

“I just wanted to take medication and so that I could live longer and take care of my children because they are still young. […] I wondered and thought an incentive being given for taking medication was a lie, because why be given an incentive when taking medication was for one’s own good? […] I don’t take medication to be given money, but to improve my health. But when the incentive is given to me, I feel happy and encouraged.”

(11494, woman, age 54)

“I had already decided to adhere to ARVs medication because I love my life so much and I still want to be alive.”

(16004, woman, age 27)

Analyses of data from women randomized to the control arm showed that similarly to women who were virally suppressed in the intervention arm, women in the control arm who were virally suppressed had overcome multiple barriers. These included severe poverty-related constraints such as food insecurity and limited money for transport to clinics. They expressed a desire for help with the costs of transport to clinic, or for drugs to be brought to their communities and homes, but were strongly intrinsically motivated to maintain their care engagement. When asked about how they came to decide to start taking ART, many discussed having simply deferred to providers who told them it was time to start, rather than consciously engaging in making a decision about it themselves. Yet these women held positive expectations that ART would prolong their lives, having witnessed others die after having refused treatment. Once on ART, adherence was reinforced through experiencing relief from HIV-related illnesses and restored health, positive social support, and good quality counseling. While they did not receive an incentive for achieving viral suppression, being virally suppressed was a reward in itself for many—they were encouraged by the results and maintained that they would continue to adhere to ART.

#### Men

The narratives of men who had been randomized to receive incentives, and were virally suppressed at follow up, were also replete with discussions of barriers that men faced and had overcome. There were gender differences in emergent themes related to these barriers. Gender role expectations for women supported their care-seeking behaviors, and attending clinic was normative for women in the setting (as one 53 year old woman [10114] told us, “women care, and most have enrolled on treatment compared to the men”), while men’s social role expectations reflected masculine gender norms that valorized men’s roles as productive workers and family providers, and discouraged care-seeking.

“Most times men leave the health treatment to the women and thus cripple the services. They stay at home and leave the women to go alone.”

(15518, man, age 40)

Compounding this, many men also reported anticipated stigma, and felt that it had inhibited their participation in testing, care-seeking and adherence. Men said that other men in the community powerfully feared blame and shame, and worried that their wives would abandon them if they tested positive:

“Most men don’t test with their wives and even when they get tested they don’t disclose to their wives. So when they come back, they want to take the medication in secrecy and through that, the medication may not be taken in time or even missed. […] They fear disclosing because they think the wife will go.”

(22863, man, age 46)

“Most still fear being known [as HIV-positive], especially the adulterous, and some pay for fake results to take to spouses… and yet they are already on treatment. And those that swap places end up lacking transport sometimes and miss treatment.”

(12095, man, age 40)

The requirements of men’s livelihood strategies and employment rules also militated against care-seeking.

“The time the doctors give me to go get treatment and care is not flexible with my work schedule. Your bosses do not expect you to leave work and go to the health unit.”

(12095, man, age 40)

These men had also overcome fears about ART, pill size and burden, and side effects:

“When I was given the tablets they were very big and I feared as if it would not pass through the throat but I accepted and swallowed them…”

(15962, man, age 50)

“When I [first] took them, I first became dizzy and started seeing things like animals that were not there, but I am okay now.”

(10993, man, age 34)

Men also discussed having started ART only after being ill.

“I did not start immediately because I was shy and feared people would know but when I got seriously sick, I was forced to go for the treatment.”

(12095, man, age 40)

Men had overcome work-related barriers to clinic attendance, and were able to successfully adhere to ART. To the extent that successful ART use supported their ability to stay productive at work and to provide for family members, especially children, it was a very powerful motivator for men’s sustained engagement in care.

“I was worried about my family because my children were still young and would not be able to fend for themselves. I worried that the worst would happen if I died. That is why I decided to take the medication in order to live longer and see my children grow.”

(15962, man, age 50)

“I like my family and health and would like to live longer and that is why I opted to be enrolled on treatment and care as required.”

(15518, man, age 40)

These men also, overall, had good social support from spouses, friends and family members to adhere to ART:

“She helps me in everything, she reminds me of the time [to take ART], and she knows I need food, a drink, full time she knows when I eat and drink.[…] She said she loved me before knowing I was sick and was not going to leave me now. We decided to stay together without any problem and we even wedded two years back.”

(22863, man, age 46)

“I usually talk to my fellow friends, especially those with the HIV virus, and we talk about staying faithful to medication.”

(10993, man, age 34)

Many of the virally suppressed men directly attributed financial incentives to their increased motivation to adhere to medications. The incentives were used to pay for transport, to offset lost income due to time spent away from work, to help pay for food, all of which supported adherence to ART. They also reported the rapid feedback of on-site VL testing and counseling was motivating.

“When you are given an incentive, you feel motivated and are able to buy something say fruits since we are advised to eat a balanced diet.”

(15962, man, age 50)

“Sometimes I would miss three days when it gets finished, when I am at the place of work or have forgotten to take it with me. But since you put incentives, I make sure I take care.[…] I was happy about it because we would be using our own money to go and get treatment or it would have taken a long time to know one’s viral load, but now, time is shortened and one knows their viral status quickly. One is able to live longer and is also able to know when to change drugs.”

(12095, man, age 40)

Yet there were also narratives that illustrated some men’s intrinsic motivation to adhere to ART:

“If I continue getting my medication then I don’t need any other motivator. I love my life and good health so I will keep taking because of that.”

(22863, man, age 46)

“I have to adhere, because I see my future bright and I would not want to miss out.”

(12095, man, age 40)

“I would have continued taking my drugs even if I did not get the incentive; it is because of my health.”

(10993, man, age 34)

Among men in the control arm who had achieved viral suppression at 24 weeks, there were expressions of strong intrinsic motivation to adhere to ART medications and stay engaged in care. Some men described having been motivated to start ART because of HIV-related illnesses, physical weakness, and poor general health that affected their ability to work and be productive. Worries about their children’s well-being was also a key motivation: one man who was worried about dying before putting his children’s “lives in order”, said, the medications “buy me time”. For others, being prompted by a clinician or wife or friend was the only cue to action they needed to enroll in care. Experiencing the positive benefits of ART—relief of symptoms, which enabled men to work, earn, and be productive—facilitated adherence and retention in care. In this somewhat older and more mature group of men, there were those who expressed a matter-of-fact acceptance of having to take ART medications for life, and a motivation to doing so because of not wanting to ‘bring the infection’ to others, and the knowledge that ART prolongs life: one man said, “I would be dead by now” if not for ART. These men also benefitted from good social support from friends, spouses, and other family members. Notably, in this group there were few mentions of having faced major barriers to ongoing engagement in care, beyond the challenge of paying for transport to clinic. There were also few mentions of having experienced poor quality of care, or of severe challenges related to medication side effects or food insecurity interfering with adherence. Rather, there were discussions of changing diet in order to tolerate medications—and receiving good counseling and clinical care that helped them to manage side effects.

### Experiences and attributes of PLHIV with detectable viral loads

#### Women

Many of the above themes related to barriers to care were also prominent in the narratives of women and men who were randomized to receive incentives, but who nonetheless had detectable viral loads (and thus did not receive the financial incentive payment at the 24-week follow up visit). Despite their detectable VL levels, these individuals had prior experience with ART, and had experienced its benefits. Women in this group were highly motivated to adhere to ART, because of their prior experiences of poor health and symptoms of HIV disease that had been relieved by taking ART.

Before I had gone to the facility, I first got a body rash and wounds and then I felt things ‘running’ through my body, but from when I started treatment, all that is gone.”

(11704, woman, age 39)

“Since then [starting ART] my health started improving, my skin got better and my eating improved. I added on some weight and did my usual duties, and I encourage women out there to test and seek treatment just like us.”

(10114, woman, age 53)

Yet, in contrast to the narratives described above, women in this group shared narratives of HIV care disruption. These instances were precipitated by poverty-related factors, namely, food insecurity and hunger. While reporting the same work-related barriers often cited by men, women discussed them in relation to food security:

“These days there is no money, we have no food and when you spend a day in someone’s plantation working, and the time for taking your drugs finds you there, you cannot leave, so you find when you have missed taking ARVs. At times when you are hungry you decide not to take any drug, because they said if you take it when you are hungry you can develop wounds inside the stomach.”

(11704, woman, age 39)

“Sometimes the jobs we do dictate the time of taking medication. Sometimes I may eat at 8.00 p.m., and other times not, depending on when I receive the money. So I cannot be sure.”

(12258, woman, age 21)

Despite the potential for financial assistance with the incentive, costs of transportation remained high for some, especially those living far from clinics. Safety was also a concern from some.

“In our community, health services are very far from us and someone has to use a motorcycle even if the person is pregnant and may even die on the way. One cannot walk when sick or heavy with baby and reach the health facility.”

(10114, woman, age 53)

“The distance from home to the clinic is long and sometimes I don’t have money for transport. I even have a child who is on care and he can’t walk from home to the clinic.”

(21641, woman, age 30)

Unlike women in the incentives arm with viral suppression, there were reports of poor treatment by providers among those with detectable VL. Prior experiences of poor quality of care discouraged these women from returning to clinic.

“We get to the health facility for treatment and we get it, but the health workers do not treat us well. You see, for us sick people, sometimes we have self-hatred and when a health worker speaks rudely to you, then one feels really bad. They speak back rudely and when one forgets to fetch medication by a day and one makes an apology, they speak rudely, blaming you, and I feel really bad… and sometimes we are chased away and one’s transport is wasted.”

(10114, woman, age 53)

“When it is not your clinic day, they can’t give you drugs; you have to wait for your day. When we go when it is not our clinic day, we are always sent back.”

(11704, woman, age 39)

Not all providers in clinics that participants opted to visit had put new ART-for-all policy guidelines into practice, and there were reports of denial of treatment:

“I have not yet started the ARVs yet though your organization after testing me said I should start them. When I did explain the doctors at the health facility, they answered back that my body’s fighting cells are still strong thus I do not need the ARVs yet. I explained that your organization said my viral load is increasing, and I [should] start ARVs, but they refused.”

(13391, woman, age 43)

Also uniquely among women in this group, there were discussions of mistrust of ART medications. One woman (age 43, 13391), for instance, said, “I was referred to [the] health center but I did not go, and I threw away the referral sheet. I did not want to start medication. What if they gave me a “pig’s drug”, how can I manage that? I refused…”

Experiences of stigma were also reported in this group. While in all groups there were some reports of unsupportive spouses, in this group were more extreme cases of enacted stigma:

“He sometimes refused to go to the health facility for treatment for three months and then stole my treatment and when I found out what had happened, I went to the counselor at the health center and told her what had happened. She helped me, gave me more with caution I took care or keep them at a friend’s and that is what I did, and kept going to my friend to get medication every evening.”

(13391, woman, age 43)

“Domestic violence is the biggest challenge that women face, whereby men can even throw away your ARVs and clinic documents when it is not yet even time for the clinic, and you fear to go back for other drugs.”

(11704, woman, age 39)

Some women did not expect to have a detectable viral load at the follow up, and expressed disappointment about the results and about not receiving the incentives.

“I felt bad and disappointed and wondered why it had increased… I tried to follow medication to the dot. But around that time, I was stressed, I had problems, school fees inclusive, but I was not happy about it.”

(10114, woman, age 53)

“I was not really bothered about money, I was more bothered about my increase of viral load, and why I couldn’t take medication just like the rest. This disappoints me and I do not like myself much at the moment because I would also feel happy when I am told that the virus is suppressed.”

(13391, woman, age 43)

Some reported feeling encouraged to continue to try to adhere. For others, multiple competing challenges in their lives meant that the potential of incentives were not on their minds. Other concerns were more pressing.

“When I had started taking ARVs, I forgot to take drugs with me for one week when I had gone to Etembe to take care of my child who was sick, and when I came back I wanted to completely drop it, but I received counseling and I resumed.”

(11704, woman, age 39)

Several women in the control arm with detectable viral loads faced barriers to care engagement that were severe in magnitude, or overlapping and synergistic. These included both anticipated stigma from husbands and experiences of enacted stigma (including at least one instance of a husband hiding ART medications), having difficulty with transport costs (especially for those living very far from clinics, for whom costs were higher than typical), and poor experiences with the health system. In this group were women who discussed having changed facilities and drug regimens, who lacked clarity about why drug regimens changed, and wished they had received better counseling.

#### Men

Men in the incentives arm who failed to achieve viral suppression at 24 weeks faced all of the above barriers, including discussions of having delayed starting ART because of fears of ART and anticipated stigma:

“I was worried about how I will get married when I am taking ARVs because whoever you would want to marry has to request for an HIV test. […] I don’t want her to know about it because once she discovers that I am taking ARVs, she can divorce me—something I don’t want.”

(15734, man, age 27)

“People were saying that when you start taking ARVs and you miss a day without taking, your health condition worsens, and you die too soon. People said that if you know that along the way you might stop taking ARVs, you better not start it. When I heard all this, I got worried.”

(22532, man, age 31)

As among their female counterparts, there were reports of health system challenges (especially long wait times) among men with detectable VL in the incentives arm. These men also reported missing ART doses because of work-related travel:

“When you go to pick medication even if the queue is long then you have to sit and wait. Whether it goes on up to 4 or 6 p.m., you will have to reschedule your other programs you may have had that day.”

(13312, man, age 52)

“You know it is hard for men to go to the clinic at 9:00 a.m. and come back home at 3:00 p.m., and because of that, some men become tired and end up going back without any drug.”

(15734, man, age 27)

“I do catering, so there is somewhere I had gone to work and took less medication, yet we still had about other four jobs booked. I still remember those are the days I missed hence the increase in viral load.”

(14234, man, age 51)

Men with suppressed VLs also discussed lack of money for food or transport as a barrier to ART adherence.

“When you are taking ARVs, it needs you to be having money all the time, because you can’t take it alone—it needs food and drinks.”

(22532, man, age 31)

“Transport at times is not there. It is a big challenge so if the medication was brought closer it would help.”

(14234, man, age 51)

They started ART late for all the reasons the ‘successful’ men did, and were similarly motivated by the promise of incentives, but were not able to overcome the forces and events that interfered with their ability to adhere to medications and keep clinic appointments.

Men in the control arm who failed to achieve viral suppression at 24 weeks spoke of coping poorly with side effects of ART medications, and some were currently experiencing them at the time of interview. Among this group were men who spoke of experiencing food insecurity, and hunger interfering with ART adherence. Like women, when men lacked enough money to pay for food, they also lacked money for transport to clinics, and it was difficult to prioritize care-seeking over a more immediate and urgent need for food. Men in this group were vocal about the need for assistance with food and transport in order to support their care engagement.

Again men, more often than women, discussed work-related challenges to care engagement, with men in particular in this group describing work-related barriers to keeping clinic appointments: due to long work hours that interfered with clinic schedules, long distances between work sites and clinics, and in one instance of a supervisor who refused to permit the participant to take time from work to make a clinic appointment, some men’s employment restricted their access to care. Among this group were men who also faced notable psychological barriers, compared to their counterparts with suppressed VL: while several men in this group described having adequate social support, and a strong motivation to try to stay engaged in care, one man reported living alone and thus having ‘no one reminding me’ to adhere to medications. Others reported severe internalized stigma and depression, including anger and self-hatred/disgust. Finally, men in this group described health systems failures and missed opportunities. One man described having stopped taking ART after symptoms subsided and he started to feel better, believing he didn’t need to continue taking them. Others described frustration at attending clinics that had long wait times or drug stockouts. Despite the many challenges faced by men in this group, they were overall very motivated to stay engaged in care and adhere to ART medications; most were knowledgeable about VL testing and described feeling motivated to ‘do better’ to adhere to medications in response to the feedback of VL test results.

## Discussion

This study has shown in granular detail the ways in which incentives can operate to facilitate actions conducive to successful engagement in care. Participants said that incentives motivated their adherence to ART and reduced missed clinic appointments due to lack of money for transport. The money was used to pay for transport, food, and to offset lost income due to time spent away from work, and allowed PLHIV to not have to make the painful choice between spending on clinic transport versus food, or risk losing employment because of absence from work. Incentives worked to activate attention and memory, as excitement about anticipating incentives helped participants to keep to their schedule for medications.

Findings also show that the influence of incentives on behavior may be contingent upon other factors. Framed positively, the psychosocial attributes that contribute to successful care engagement, even without incentives, include intrinsic motivation to adhere to ART, and positive expectancies about the efficacy of ART (gained through seeing others benefit from ART, seeing the negative consequences of others not taking ART, and one’s own experiences of the benefits of ART use). The interpersonal attributes and circumstances conducive to ART uptake and adherence include a supportive spouse or partner as well as social support in friend and family networks. Structural conditions under which incentives may not be needed include adequate money for food and transport to clinic, good quality services in the health system (being treated promptly and politely by providers, and receiving good counseling, appropriate clinical regimens, and management of side effects), and flexible working conditions that permit travel to clinic for appointments. The use of incentives for certain non-suppressed persons (for example, those with challenges affording transport, or with food insecurity) may be helpful, whereas for others (e.g., who are food secure, and can afford transport), barriers may lie elsewhere. In sum, the findings suggest that incentives may need to be combined with other interventions in order to help PLHIV overcome the full spectrum of barriers to achieving viral suppression.

This study is subject to some limitations: the analyses were cross-sectional and did not permit within-case observation of change over time. The data collected, thus retrospective, were subject to recall bias, and particularly social desirability bias towards intervention if participants assumed that researchers wanted them to attribute positive outcomes to incentives. However, the rigor of our design was strengthened by interviewing ‘successes’ and ‘failures’ in both intervention and control arms.

We have noted that the trial in which this qualitative study was embedded, incentives had no significant effect on achieving or sustaining viral suppression at 24 weeks [14], joining three other financial incentives intervention studies among PLHIV that sought, and failed, to increase viral suppression rates [15]. Authors noted several potential reasons for the non-significant effect of incentives on viral suppression, beginning with the already high viral suppression rates in the control and intervention groups at baseline. They noted that while it may be speculated that incentives could be more effective in low-income populations with low ART adherence or low viral suppression, subgroup analysis showing that participants unsuppressed at baseline did not respond to financial incentives did not support this hypothesis. In a commentary, Thirumurthy and colleagues [15] have suggested refinements to the way incentives are designed and implemented may better address barriers to care engagement and adherence; providing immediate rewards as well as increasing frequency, framing incentives as losses rather than gains, and delivering incentives to clinician/patient pairs or teams, are strategies worthy of consideration. Indeed, one recent randomized trial found a significant impact of financial incentives on retention in HIV care and viral suppression among adults starting ART in Tanzania [16]. However, the findings of this qualitative study support the observation from the HPTN 065 trial that for some, “financial incentives may be insufficient to overcome strong social or structural barriers, and unnecessary for those intrinsically committed to remaining adherent.” [17].

This study confirms prior research showing multiple domains of influence on care engagement and adherence in a resource-poor setting [18–23], with distinct gender differences in the salient factors that militate against successful care engagement. We observed differences across the narratives of participants who did, and who did not, achieve viral suppression: participants randomized to receive incentives but with detectable VL faced a wider range, complexity and severity of challenges, compared to those with viral suppression: participants described HIV care disruption, precipitated by poverty-related factors such as food insecurity and lack of money for transport (especially in those living far from clinics), and prior experiences of poor treatment by providers. Among women in particular there were narratives of multiple compounding barriers (e.g., needing to travel to care for sick children or other family members) along with severe forms of stigma (e.g., intimate partner violence, withholding of medications). Among men in particular, internalized stigma, fears of ART, and poorly managed side effects, and work-related barriers (making long wait times at clinics especially intolerable) interfered with ART adherence and keeping appointments. These accounts support the sense that practical interference in patients’ daily lives can overrule their behavioral intention, supporting Ware’s observation of the temporal nature of care engagement, and her notion that “missed visits and ensuing reluctance to return over time erode patients’ subjective sense of connectedness to care” [23]. In short, while helpful, the incentives were not sufficient to help these PLHIV overcome what was sometimes a cascading set of challenges.

Among these well-recognized challenges are health systems failures and missed opportunities that must be addressed in order for PLHIV to successfully engage in care. Our findings reflect a heterogeneity in care provision contexts. The standard of care in this setting at the time of this study was universal ART, but not all providers were necessarily on board with this new policy; the study team did not dictate where people would seek care, nor did it collect information about where participants sought care. Yet it was clear that health systems interventions remain needed to offer what patients experience to be good quality care: supportive counseling, and management of side effects provided by nonjudgmental and friendly providers. Severe poverty-related constraints such as food insecurity and limited money for transport to clinics, especially for the poorest of the poor and those living remote from clinics, could be directly addressed through broader initiatives to improve income and livelihoods. Such initiatives would also support what underlies many PLHIV’s strong intrinsic motivation for care engagement: aspirations for a future healthy life, and desire to support one’s children’s healthy transitions to adulthood. Being seen to work productively and to be a successful provider is a powerful motivator for men’s care engagement; policy interventions to remove workplace or employment-based barriers to clinic attendance, even and especially in informal sector work, may help to facilitate broader changes in gender norms that are conducive to men’s care seeking.

## Conclusions

In considering why incentives sometimes fail to achieve behavior change, it may be helpful to attend to the full set of factors- psychological, interpersonal, social and structural- that militate against the behavior change required to achieve behavioral outcomes. To be effective, incentives may need to be combined with other interventions to address the spectrum of barriers to HIV care engagement.
